# Small Bowel Obstruction in Postpartum Vaginal Delivery due to Prior Abdominal Adhesions Case Report

**DOI:** 10.1155/2023/6563205

**Published:** 2023-03-28

**Authors:** Liubin Yang, Lydia Kao

**Affiliations:** Baylor College of Medicine, 1 Baylor Plaza, BCM 610, Houston, TX 77030, USA

## Abstract

Intestinal obstruction rarely occurs after uncomplicated vaginal deliveries. Here, we present a case of a multiparous woman with a history of prior appendectomy presenting with generalized, nonspecific abdominal pain that was out of proportion to exam findings. Initial abdominal X-ray was nonspecific, and subsequent computed tomography (CT) abdomen showed closed small bowel obstruction requiring surgical repair. We present a case of intestinal obstruction occurring within 24 hours of uncomplicated vaginal delivery with a risk factor of a prior appendectomy surgery and the use of CT abdomen and pelvis to expedite diagnose.

## 1. Introduction

Intestinal obstruction is rarely observed after an uncomplicated vaginal delivery with an incidence of 1 in 3000 and a high mortality rate of 18–25% [1]. The differential diagnosis for acute abdominal pain in the postpartum period includes postoperative complications including hemoperitoneum, appendicitis, cholecystitis, adhesive intestinal obstruction, ileus after Cesarean section, perforated peptic ulcer, bladder rupture [2], bacterial peritonitis [3], and ovarian torsion [4]. Specifically related to postpartum is Ogilvie's syndrome, which is denervation of parasympathetic nerves causing atonic distal colon and pseudo-colonic obstructions. This usually occurs after Cesarean section [5], trauma, pelvic surgery, sepsis [6], and a few cases of vaginal delivery [7]. Idiopathic intussusception has also been reported during the postpartum period following vaginal delivery [8]. Curiously, intestinal ileus is common in other mammals including intestinal pseudo-obstruction observed in lactating mice in the second week postpartum [9, 10] and also in postpartum mares [11]. Therefore, acute abdomen after delivery has a wide differential diagnosis and should be triaged promptly to avoid the high rates of maternal mortality.

## 2. Case Presentation

**Figure 1:**
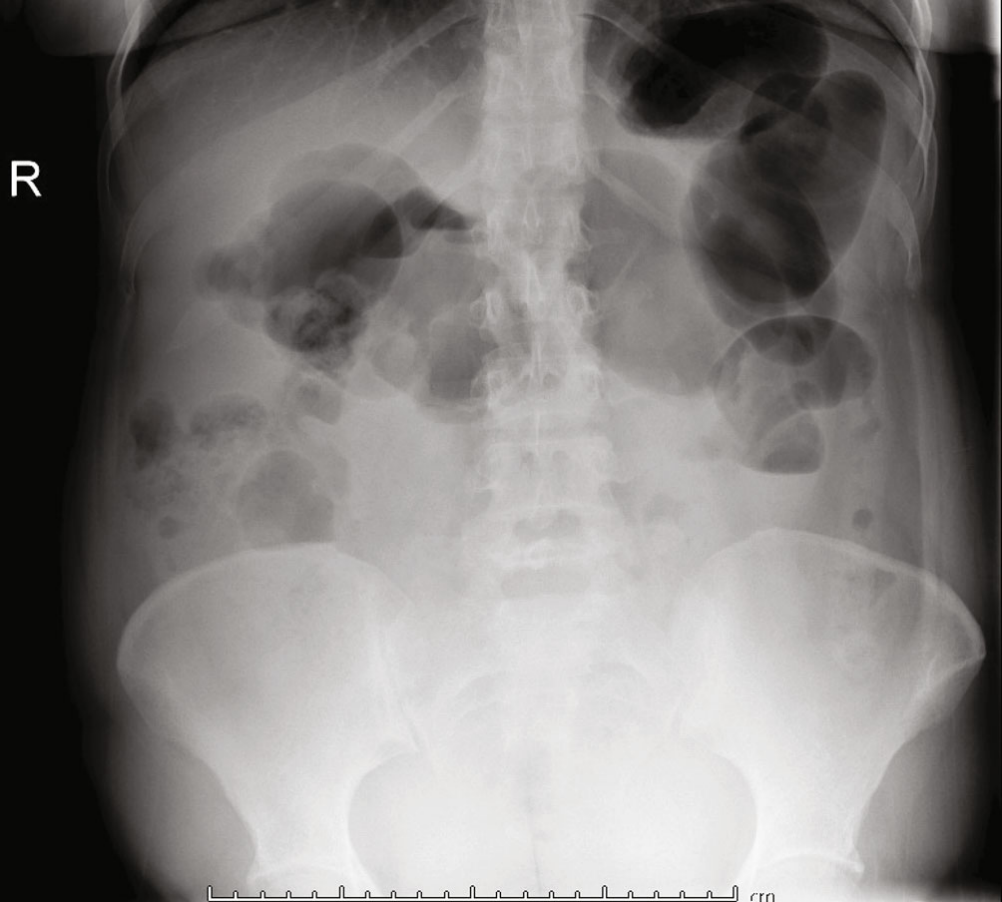
Image of abdominal X-ray showing mildly distended dilated gas-filled large bowel without gas in the rectum due to ileus or early obstruction.

**Figure 2:**
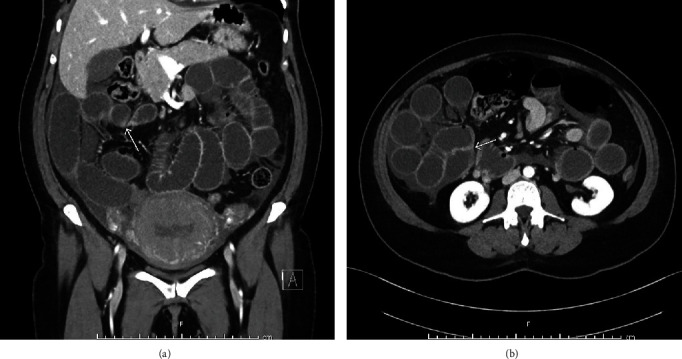
(a) Coronal view of CT of abdomen and pelvis with intravenous contrast showing dilated loops of bowel, closed loop small bowel obstruction with transition point (arrow) in the right mid abdomen. (b) Axial view of CT of abdomen and pelvis with intravenous contrast showing dilated loops of bowel, closed loop small bowel obstruction with transition point (arrow) in the right mid abdomen.

This patient is a 41-year-old multiparous female who presented with painful contractions with 2 cm cervical dilation and admitted for augmentation of labor. Her medical history was uncomplicated. Her surgical history was significant for open appendectomy at age 12 years and a dilation and curettage at age 27 years for a molar pregnancy. She has had five previous spontaneous vaginal deliveries and no Cesarean sections. She had an uncomplicated vaginal delivery 17 hours after admission with no lacerations. She received 0.2 mg of methylergonovine and 800 *μ*g of misoprostol for uterine atony during delivery with total blood loss of 400 mL. On postpartum day 1, she tolerated a full breakfast without nausea or vomiting, urinated spontaneously, and was ambulatory. About 11 hours after delivery, she had new onset generalized abdominal pain described as cramping and severe. Her vitals were as follows: heart rate 80 bpm, blood pressure 147/80 mm Hg, oxygen saturation 100% on room air, respiratory rate 18 breaths per minute, and blood glucose 98 mg/dL. She had a relatively mild abdominal examination with moderate tenderness in all quadrants without rebound or guarding. Bedside, abdominal ultrasound showed thin endometrial stripe, uterus well-contracted, and without abdominal free fluid. Her hemoglobin was 11.7 g/dL. She was initially given a mixture of aluminum–magnesium hydroxide, calcium carbonate, soft diet, and pain medications with symptomatic improvement. However, the next day, she developed oral intolerance with persistent abdominal pain. Her electrolytes, l iver function tests, amylase, lipase were within normal limits. Her white blood cell count was 15.2 × 10^9^/L. Abdominal X-ray showed mildly distended dilated gas-filled large bowel without gas in the rectum possibly due to ileus or early obstruction (Figure 1). Subsequent computed tomography (CT) abdomen and pelvis revealed closed loop small bowel obstruction with transition points in the right mid abdomen (Figure 2). The distal jejunum appeared dilated up to 3.6 cm in diameter. The terminal ileum and the distal ileum, as well as the duodenum and the proximal jejunum were not dilated. She underwent an urgent diagnostic laparoscopy with findings of dusky small bowel and conversion to laparotomy. Intra operative findings included dusky appearing small bowel and bowel adhesions. As seen on imaging, there was a displacement of the cecum towards the midline causing some degree of malrotation. Intraoperatively, the cecum was noted to be located medially due to an adhesion between the cecum and the omentum, which was lysed. There were also adhesions between the small bowel mesentery and the uterus that were lysed, which freed the small bowel that was entrapped between the uterus and the small bowel mesentery. Given the location of the adhesions near the cecum, which is in close proximity to the appendix that was removed previously, the adhesions were mostly likely acquired post appendectomy. No internal herniations were noted intra-operatively. The adhesive band between the uterus and the small bowel mesentery, which were lysed, likely the cause of the bowel obstruction. After lysis of adhesions, the small bowel was noted to be viable. Post-operatively, she had high-volume nasogastric tube output until post-operative day 4 when her nasogastric tube was removed, and her diet was advanced, and she was discharged home.

## 3. Discussion

We present a rare case of intestinal obstruction within 24 hours of vaginal delivery. Intestinal obstruction has been reported in a few case reports in the 1960s following vaginal deliveries [1, 12, 13] with incidence of 1 in 3000 vaginal deliveries and initial mortality rate of 18–25% due to misdiagnosis or delays in diagnosis [12].

It is best to have high index of suspicion for obstruction if the patient has risk factors, such as uterine fibroid seen on ultrasound during pregnancy [14] or other structural abnormalities including paraduodenal hernia [15], or a prior history of abdominal surgery. In this case, the patient had a remote history of open appendectomy at age 12 years. Though rare, open appendectomy in children can be associated with adhesion-related bowel obstruction in 1.9% of the time [16]. We had high suspicion because of her risk factors and worsening status, which prompted us to order diagnostic imaging. However, the diagnosis was difficult to make because of the nonspecific nature of postpartum abdominal complaints [17]. It is possible that with the rapidly decreasing size of the uterus after delivery, both the adhesions attachment points and bowel loops shifted to ensnare the loop of bowel.

The best modern imaging diagnostic technique in the postpartum period in the context of a challenging diagnosis is CT of the abdomen and pelvis. Relying other imaging may delay care, such as in another case report of a postpartum small bowel obstruction diagnosed 17 days postpartum following insidious onset from postpartum day 1 [18]. In that case, the diagnostic imaging initially used was ultrasound, which showed free fluid, but the diagnosis of obstruction was revealed subsequently with CT abdomen and pelvis. The sensitivity of detecting a small bowel obstruction in abdominal radiography varies from 59 to 93% with specificity of 83%, whereas CT scan has a sensitivity of 64% and specificity of 79–93% [19–21]. However, if the differential includes other abdominal pathology, such as volvulus, the specificity with abdominal X-ray decreases to 60% [22]. Furthermore, CT imaging provides details not found on X-ray including transition points, severity of obstruction, and etiology of the obstruction, such as masses or hernias [23]. Given advancements in diagnostic imaging in the past 40 years, it would be more useful to start with CT abdomen, popularized since the 1980s [24], to diagnose acute abdominal processes in the postpartum period for patients with nonspecific complaints. The radiation risk is relatively low. It would take 870 routine abdominal CT with contrast for women at age 40 years to develop one radiation-induced cancer (rate of 0.1%) [25] compared with the high mortality rate of 18–25% postpartum bowel obstruction. Therefore, CT of the abdomen and pelvis may be a better initial imaging modality to triage non-specific acute, severe abdominal complaints.

In conclusion, we present a case of closed-loop small bowel obstruction that manifested after a vaginal delivery. Given the diagnostic challenges of non-specific severe abdominal pain after a vaginal delivery, a CT was critical in the diagnosis, which led to a timely surgical treatment. The obstruction was successfully relieved surgically without the need for bowel resection. Therefore, a high index of suspicion should be maintained for severe acute abdominal pain after vaginal delivery especially if the patient has had any abdominal surgery.

## Additional Points

1. Maintain a high index of suspicion for non-obstetrical causes of acute abdominal pain in the postpartum period especially in patients with prior abdominal surgery.
2. Prompt evaluation with CT abdomen and pelvis is valuable to help diagnose abdominal pathology in the postpartum period.
